# Sequence specific sorting of DNA molecules with FACS using 3dPCR

**DOI:** 10.1038/srep39385

**Published:** 2017-01-04

**Authors:** David J. Sukovich, Shea T. Lance, Adam R. Abate

**Affiliations:** 1 Bioengineering and Therapeutic Sciences, University of California San Francisco, San Francisco, California, USA; 2 California Institute for Quantitative Biosciences, University of California San Francisco, San Francisco, California, USA; 3 UC Berkeley-UCSF Graduate Program in Bioengineering, University of California San Francisco, San Francisco, CA, USA

## Abstract

Genetic heterogeneity is an important feature of many biological systems, but introduces technical challenges to their characterization. Even with the best modern instruments, only a small fraction of DNA molecules present in a sample can be read, and they are recovered in the form of short, hundred-base reads. In this paper, we introduce 3dPCR, a method to sort DNA molecules with sequence specificity. 3dPCR allows heterogeneous populations of DNA to be sorted to recover long targets for deep sequencing. It is valuable whenever a target sequence is rare in a mixed population, such as for characterizing mutations in heterogeneous cancer cell populations or identifying cells containing a specific genetic sequence or infected with a target virus.

Heterogeneity is an important aspect of many biological systems, impacting their ability to respond to stimuli or evolve against evolutionary stresses. However, the massive heterogeneity present in most systems also imposes technical challenges to their characterization. A single ocean sample, for example, can contain billions of microbes, each with its own genes, gene clusters, and genomic structure^1^. Moreover, even the highest throughput modern sequencers are capable of sampling just a minute fraction of the sequences present in most systems, and the “reads” are obtained in the form of short, ~100 base fragments^2^,^3^,^4^. Genetic structures like genes, gene clusters, and genomes typically span much longer scales, from kilobases to gigabases of sequence^5^,^6^,^7^,^8^. To extract meaningful biological insight from the short read data requires “assembling” reads into “contigs” spanning the relevant genetic length scale. Here, however, the shortness of the reads, sparseness of the sampling, and massive diversity of the system impose technical challenges, making assembly difficult and error-prone^4^,^9^. Often, useful contigs can only be generated for the most abundant organisms, and the overwhelming majority of species present at low and moderate levels are missed^10^.

An effective means to overcome the challenges introduced by short read data is to enrich for target sequences prior to sequencing^11^. This can greatly improve assemblies and has been used to address questions ranging from deconvoluting cancer heterogeneity^12^ to finding gene clusters encoding new, useful molecules in the microbiome^13^. A common enrichment strategy is PCR, in which primers specifically amplify a target sequence from a heterogeneous mixture. However, PCR methods can introduce artifacts^14^,^15^, yield non-specific products^16^, and are limited to enriching for sequences up to ~10 kb. Digital droplet PCR (ddPCR) overcomes some of these issues and can be multiplexed to recover thousands of targets using microfluidics^17^,^18^, but since only amplicons are sequenced, it is still limited to enrichment lengths of a few kilobases^19^,^20^. A modification of this technique has been reported where nucleotides are detected and sorted using microfluidic techniques, but is further hampered by the need to use custom-built machinery for the sorting of these molecules^17^. An entirely different enrichment strategy is to physically purify molecules using beads coated in capture probes and sorting them using flow cytometry^21^; however, this approach also is limited to targets of a few kilobases per capture probe, necessitating thousands of probes to recover megabases of sequence^22^. An optimal system for enriching DNA would significantly increase the lengths of molecules that could be recovered per target probe.

In this paper, we introduce double emulsion digital droplet PCR (3dPCR), a method to physically sort long target molecules out of a heterogeneous population with FACS. Whereas PCR and oligo-capture methods are limited to a few kilobases of sequenc e length recovered, 3dPCR can theoretically exceed 100 kilobases. In addition, the PCR identification can be multiplexed, to recover molecules with multiple, distinct sequences – a new capability not possible with PCR or oligo methods that is useful for differentiating between organisms with combinatorial genetic diversity, such as microbes, viruses, and cancer cells.

## Results

3dPCR is a cousin of common ddPCR, except that rather than performing single-molecule PCR assays in water-in-oil single emulsion droplets, it performs the reactions in double emulsion droplets. The benefit of this is that double emulsions, unlike single emulsions, are suspended in an aqueous carrier phase, allowing them to be read and sorted using common flow cytometry instruments^23^. Using specific PCR reactions performed in each droplet, each molecule in the sample is “read” at a specific location to determine if it is a target molecule. If so, the PCR assay yields a fluorescent signal that fills the encapsulating droplet, allowing it to be detected and recovered by FACS sorting. Using TaqMan PCR with probes of different color, the method can be multiplexed, to differentiate between molecules that share partial homology and sort based on combinatorial rules, such as a given molecule containing two specific sequences.

**Figure 1:**
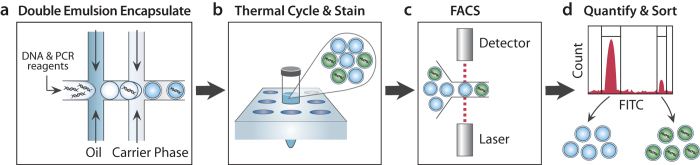
Overview of 3dPCR workflow. A heterogeneous sample of DNA is encapsulated in double emulsion droplets microfluidically (**a**) and thermal cycled (**b**). Droplets containing the target sequence undergo amplification, producing a fluorescent signal that can be detected on FACS (**c**) and used for quantitation and sorting (**d**).

The objective of 3dPCR is to enable the detection, quantitation, and isolation of specific DNA molecules in a heterogeneous sample. To accomplish this, 3dPCR encapsulates DNA from a sample into double emulsion droplets (Fig. 1a) and performs a specific PCR reaction in every droplet interrogating for target sequences (Fig. 1b). If the sequences are present, PCR amplification occurs, generating a fluorescent signal that can be detected on FACS (Fig. 1c) and used to sort and recover the target sequences (Fig. 1d). Sorted droplets can be pooled and analyzed as a mixture, or dispensed singly into wells. While we use standard PCR techniques for detecting amplification, with SYBR or TaqMan fluorescence as the readout, other reactions and readouts are applicable, including isothermal amplification (MDA, LAMP, RPA) and probes (molecular beacons, scorpion probes).

### 3dPCR double emulsions

**Figure 2:**
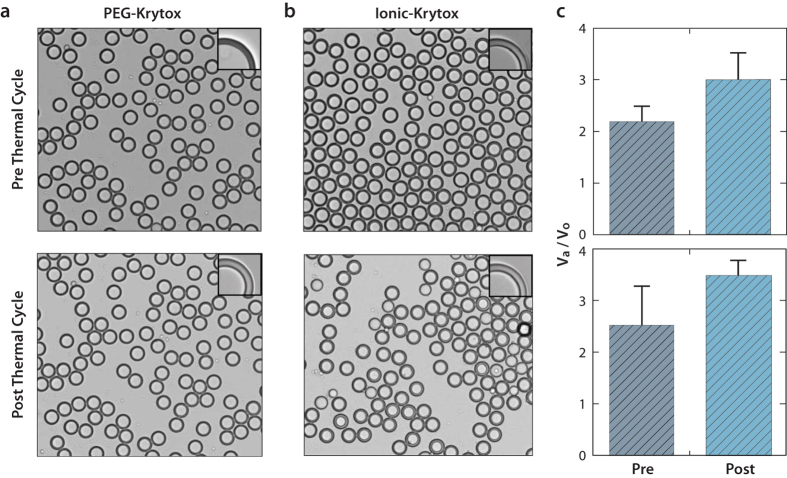
Double emulsion monodispersity. We form double emulsions microfluidically, which are stabilized by either a non-ionic PEG-Krytox (**a**) or ionic-Krytox surfactant (**b**). Both yield extremely stable droplets that survive thermal cycling. However the droplets swell during cycling to balance the osmolality of the inner core of the droplet with the miscible aqueous carrier (**c**) (Pre = pre thermal cycling; Post = post thermal cycling; V_a_ = volume of the aqueous phase of the double emulsion; V_o_ = volume of the oil phase of the double emulsion).

3dPCR is made possible by the ability of microfluidic devices to form monodisperse droplets from a sample containing a mixture of DNA molecules. By controlling the concentration of molecules in the sample, the number encapsulated in each droplet can be tuned down to single molecules^24^. Critical to using double emulsions for PCR detection of target molecules is stabilizing the droplets through thermal cycling reactions. In this work we accomplish this using fluorinated oils with fluorosurfactants due to the incredible stability of the emulsions they form, including for the high temperatures of PCR. We use two surfactants depending on the PCR assay conducted in the droplets: a non-ionic polyethylene glycol (PEG) Krytox surfactant, and an ionic-Krytox surfactant (Fig. 2).

The non-ionic surfactant comprises a PEG head group covalently bound to two fluorinated block copolymer tails; the PEG is soluble in water, while the fluorinated tails are soluble in the oil phase of our emulsions^25^. When this amphiphilic molecule adsorbs to the water-oil interface of a droplet, the PEG head group coats the inner-surface of the water droplet. PEG is effective at preventing the adsorption of proteins to hydrophobic interfaces, which is important for maintaining the enzymes in the bulk of the droplet, where they can carry out PCR. To detect the amplification products of the reactions, we add SYBR Green dye to the carrier phase after amplification. SYBR Green becomes fluorescent when intercalated into DNA^26^ and readily partitions through the thin oil shell of the double emulsions, staining the positive droplets and making them detectable via FACS. The double emulsions formed with this surfactant are monodisperse and survive PCR thermal cycling (Fig. 2a).

While the non-ionic surfactant provides good stability and enables efficient PCR in double emulsions, the shells are leaky to dye molecules; indeed, we exploit this to stain positive droplets with SYBR post-thermal cycling. However, this leakiness also creates a challenge when performing TaqMan PCR in the droplets: the reaction uses DNA probes labeled with a fluorescent dye and quencher; upon amplification, the probe is cleaved releasing the dye to fluoresce. However, the untethered dye can readily partition through the double emulsion shell, leaking out of the droplets and resulting in loss of the TaqMan signal.

To rescue the signal, we must contain the dye, which we achieve by introducing a barrier to its partitioning through the shell. We switch to the ionic-Krytox surfactant which, in contrast to the PEG surfactant, readily adsorbs proteins to the droplet interface^25^. This would normally be detrimental to PCR, as it would adsorb the polymerases, but we also include bovine serum albumin (BSA) in the droplet at high concentration. BSA is often added to PCR to increase efficiency of the reaction^27^ and, being a protein, is readily adsorbed to the interface of ionic-Krytox stabilized droplets^28^,^29^,^30^. At the interface, it forms a “skin” that prevents further adsorption of proteins and provides a barrier to partitioning of the freed dye molecules^30^. This makes TaqMan positive droplets detectable on FACS. This surfactant also forms monodisperse double emulsion droplets stable to thermal cycling (Fig. 2b).

The double emulsion droplets, regardless of the surfactant used, swell during PCR (Fig. 2c). We believe this to be due to partitioning of water and buffer molecules through the shells, to balance the osmolarities of the inner and outer aqueous phases. Shell permeability must thus be carefully considered when performing reactions in double emulsion droplets, but also provides a facile means of modulating droplet contents by adding compounds to the carrier phase.

### One-color 3dPCR with SYBR readout

**Figure 3:**
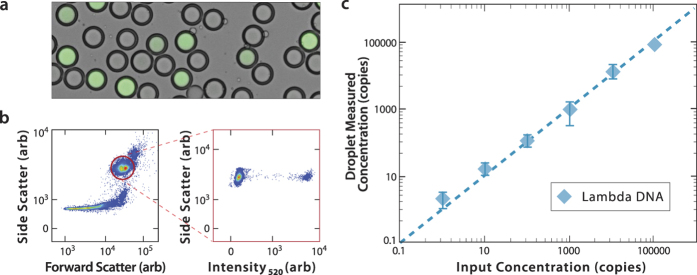
3dPCR with a one-color SYBR assay. Lambda virus DNA is mixed with primers targeting the virus and PCR reagents, formed into double emulsions, thermally cycled, and stained with SYBR green (**a**). The droplets are processed through FACS, gated on scattering to discard all non-single-core double emulsion events, the remainder for which are plotted for fluorescence values (**b**). Six samples with different Lambda virus concentrations are processed and quantified, demonstrating that, as expected, the proportion of fluorescent droplets scales with the Lambda virus input concentration, in accordance with Poisson encapsulation statistics (**c**).

3dPCR, like ddPCR, can be used to count target DNA molecules in a heterogeneous mixture. To demonstrate this, we analyze samples of Lambda virus at different concentrations (Fig. 3). We spike Lambda DNA into PCR mix with primers targeting a portion of the Lambda genome (35,515–35,664 bp). We double-emulsify and thermal cycle the sample, then add SYBR to the carrier phase to stain the positive droplets (Fig. 3a). To analyze the ~5 million double emulsion droplets, we use a flow cytometer. The double emulsions are monodisperse and large compared to cells (~40 μm) and, thus, appear as a tight cloud at high intensity in the forward and side scatter channels (Fig. 3b, *left*). Debris appear at lower scatter events and oil droplets resulting from ruptured double emulsions as a distribution of low side- and high forward- scatter events. Large, multicore double emulsions are occasionally produced by the microfluidic drop maker and appear as a relatively narrow and very high side- and forward-scatter population. Based on the counts of the events for the side- and forward-scatter populations, about 75% survive thermal cycling and FACS detection. To analyze the single-core double emulsion population, we gate the corresponding cloud in the scatter channels (red circle), and plot the fluorescence of this subpopulation (Fig. 3b, *right*). We observe two populations in the fluorescence channel, a dim one corresponding to negative droplets devoid of the target, and a bright one corresponding to droplets containing the target. To measure the target concentration in the sample, we calculate the proportion of dim and bright droplets, normalizing by volume to convert to concentration units. We perform this analysis on six samples varying in Lambda virus concentration by ~5 orders-of-magnitude and find that, as expected, the fraction of bright droplets scales in proportion to the input concentration (Fig. 3c).

### Multiplexed 3dPCR with a TaqMan readout

**Figure 4:**
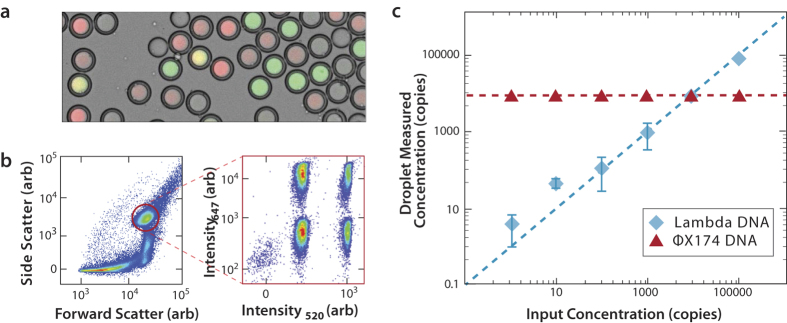
3dPCR with a two-color TaqMan assay. Lambda- and ϕX174 virus DNA are mixed together and combined with primers and TaqMan probes targeting both viruses. The samples are formed into double emulsions and thermal cycled (**a**). The droplets are processed through FACS, gated on scattering to discard all non-single-core double emulsion events, the remainder for which are plotted for fluorescence values (**b**). Four fluorescent populations are observed, corresponding to the four possible combinations of Lambda and ϕX174 virus encapsulation. Six samples with different Lambda virus concentrations with constant ϕX174 virus conditions are processed, and quantified, demonstrating that, as expected, the proportion of ϕX174 positive droplets is unchanged between samples, but that of Lambda virus scales with the input concentration, in accordance with Poisson encapsulation statistics (Dashed curve) (**c**).

TaqMan PCR increases the specificity of conventional PCR by using a TaqMan probe to generate the fluorescence readout. Only if the amplification products are homologous to the TaqMan probe sequence will the fluorescent signal be generated, reducing false positives due to non-specific amplification^31^. TaqMan PCR also allows the reaction to be multiplexed by using dyes of different color to label the probes, to interrogate for multiple DNA sequences in the sample simultaneously. This allows us, for example, to distinguish between droplets containing a single sequence and ones containing multiple sequences. To illustrate this, we spike Lambda virus genomes into a sample of ΦX174 virus genomes, varying the concentration of Lambda virus over ~5 orders-of-magnitude while holding that of ΦX174 fixed. We analyze the sample with 3dPCR using TaqMan assays targeting Lambda (green dye) and ΦX174 (red dye) virus (Fig. 4a). The Lambda and ΦX174 genomes are encapsulated randomly in the droplets, so that we expect four populations, droplets devoid of both targets (dim), with one target (pure green or red), and with both targets (yellow).

We analyze the droplets with FACS and again gate the scatter population corresponding to the double emulsions (Fig. 4b, *left*). We plot the red and green fluorescence of this subpopulation and observe the expected four groups (Fig. 4b, *right*). Because the TaqMan assays yield “binary” signals in which the positive droplets cluster together and do not overlap with the negative droplets, the number of events detected in each cluster is insensitive to the exact position of the gating thresholds. Just as with the one-color experiment, the proportion of positive and negative droplets falling within the different populations can be used to estimate the concentrations of the different viral genomes in the mixed sample, over a wide dynamic range (Fig. 4c). This shows that 3dPCR with a TaqMan readout can quantitate multiple DNA species in a sample simultaneously. Moreover, it allows specific instances in which two different targets are present within the same droplet to be identified (yellow droplets, Fig. 4a). This is not possible with conventional qPCR and is useful for correlating different sequences together, for example, to characterize the lengths of DNA molecules based on the presence of sequences at defined distances from one another^32^,^33^ to characterize recombination events based on the presence of different sequence combinations within a single molecule^34^, and to characterize combinatorial associations of distinct molecules within the same entity, such as the DNA of viruses with segmented genomes^35^,^36^ or the probability of a specific pathogen to infect a given host. Such associations, normally, are only measurable with DNA sequencing, but 3dPCR provides a rapid and inexpensive alternative

### Sorting single DNA molecules with FACS

**Figure 5:**
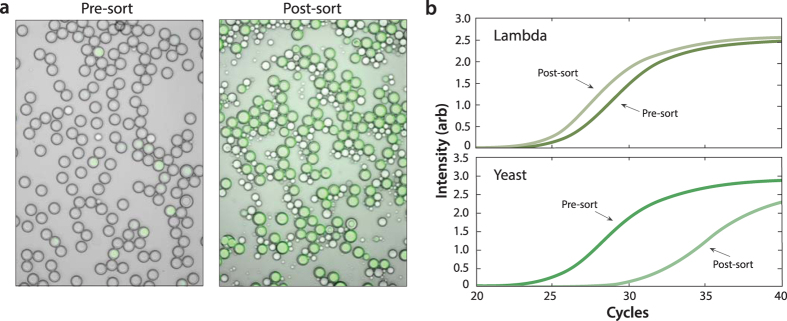
Enrichment of Lambda virus DNA from a background of *S. cerevisiae* (yeast) genomic DNA by sorting the molecules with FACS. Lambda DNA is spiked into *S. cerevisiae* genomic DNA at 1:100 concentration, and processed through (**a**) 3dPCR (left) and FACS (right). Some double emulsions pop during FACS, leaving behind small oil droplets in the sorted population. We quantify enrichment using qPCR with primers targeting a region of the *S. cerevisiae* genome and a different region of the Lambda genome that was amplified in the 3dPCR reaction. Based on the curve shifts, we estimate that the ratio of Lambda-to-*S. cerevisiae* DNA increases by 83 fold by sorting (**b**). Brightfield and fluorescence are overlaid in these images.

3dPCR allows a FACS instrument to “read” a target DNA sequence using a PCR assay. However, FACS is capable of more than detection, it can also sort based on the measurement. This, in essence, allows a mixed population of DNA molecules to be interrogated individually, to recover all molecules matching the PCR assay criterion; it also provides a novel way to enrich DNA that affords substantial advantages over conventional methods based on common PCR or oligo capture. To demonstrate this ability, we sort DNA samples using 3dPCR. We spike Lambda virus DNA into a background of *S. cerevisiae* genomic DNA, and sort the sample with TaqMan probes targeting Lambda (Fig. 5a, *left*). The sorted double emulsion droplets are TaqMan positive, although there are also many oil droplets (Fig. 5a, *right*). The oil droplets are remnants of double emulsions that explode during FACS detection, ruptured by the shear rates generated by the sheath flow spacing and the forces generated by sorting. Based on the scatter population sizes and imaging of sorted droplets, we estimate ~40% of the double emulsions survive through the PCR and FACS steps. Double emulsion rupture during FACS can be mitigated by reducing double emulsion size, increasing the nozzle size of the FACS, and processing the droplets slower.

To confirm the enrichment achieved by sorting the double emulsions, we use qPCR, analyzing the sorted and unsorted samples with primers targeting a different region of the Lambda genome and a region of the *S. cerevisiae* genome (Fig. 5b). The Lambda virus primers amplify slightly sooner in the sorted than the unsorted sample (Fig. 5b, top), even though the total amount of DNA is the same, indicating that sorting increases the number of Lambda virus genomes. Conversely, the *S. cerevisiae* primers amplify much later in the sorted than in the unsorted sample, indicating that *S. cerevisiae* DNA has been strongly de-enriched by sorting (Fig. 5b, bottom). Based on the qPCR curve shifts, we estimate the ratio of Lambda-to-*S. cerevisiae* DNA has changed by a factor of 83; this is close to the theoretical expectation based on Poisson encapsulation of the molecules for our loading rate of 1% Lambda-positive droplets. Higher enrichment ratios can be achieved by diluting the sample further, reducing the amount of *S. cerevisiae* DNA encapsulated in the positive droplets; however, this also reduces the frequency of positive droplets, necessitating more sorting to recover the same number of positive events. This demonstrates that 3dPCR is an effective means of sorting target DNA out of a mixed sample with FACS.

### Size distribution of sorted DNA

**Figure 6:**
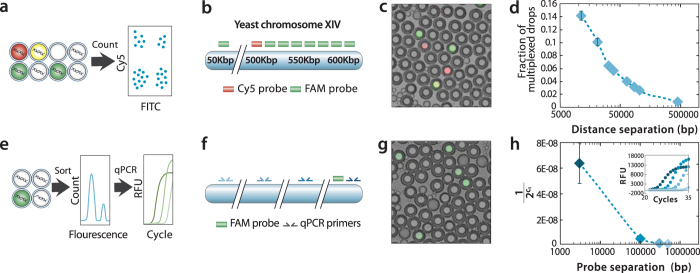
Size distribution of encapsulated DNA using 3dPCR. *S. cervisiae* (yeast) genomic DNA is encapsulated in double emulsions with Cy5 primer/probe and FAM primer/probe sets (**a**). The primer/probe sets are separated by 10 Kbp to 500 Kbp along the yeast genome (**b**). After thermal cycling, the droplets are imaged (**c**) and fluorescence intensities measured with flow cytometry. As the distance between probes increases, the fraction of double-positive droplets decreases, indicating that long molecules are rarer than short molecules due to fragmentation of the DNA (**d**). To confirm this, we sort the DNA with a single FAM probe and interrogate molecule length with qPCR (**e**) with secondary probes at increasing distances from the sort probe (**f**). After thermal cycling, sparse positive droplets are evident (**g**) which are recovered and analyzed (**h**). The sorting with qPCR confirms the multiplexed results showing that long molecules are rarer than short ones, but that molecules up to 500 Kbp are present in the sample. These molecules can be recovered by sorting using a multiplexed TaqMan assay targeting the two extremes of this region.

An advantage of 3dPCR over other DNA enrichment methods is that it allows molecules >100 Kbp in length to be recovered using just ~100 bp of TaqMan identifying sequence. To illustrate this, we measure the lengths of molecules detected and recovered via 3dPCR with a multiplexed assay. We include in each droplet red (Cy5) and green (FAM) TaqMan probes targeting yeast chromosome XIV at defined distances from one another. If a droplet contains a DNA molecule shorter than the separation distance of the probes, the droplets will be either pure red or green (Fig. 6a, left). If, however, the fragment is longer than the separation distance, the droplet may be double positive (yellow), allowing us to estimate the fraction of molecules in the sample that are above the separation length by counting the number of single- or double-positive events via flow cytometry (Fig. 6a, right). By repeating the experiment for red and green probe pairs at increasing separation (Fig. 6b), we can estimate the length distribution of molecules in the sample.

Post thermal cycling, the droplets obtain a fluorescence that depends on the number of target sequences they contain, as shown by the representative image in Fig. 6c. By repeating this for all probe pairs, we find that the fraction of double-positives is inversely proportional to the separation distance between the probes (Fig. 6d). This indicates that long molecules are present, but they are rarer than short molecules. Based on the 3dPCR fractions, we estimate that 3% of molecules are >100 Kbp in length.

As an additional confirmation of the lengths of molecules obtained by 3dPCR, we perform an experiment in which we use a single probe to detect yeast chromosome XIV, and FACS to recover these molecules, which are then interrogated via qPCR (Fig. 6e). The detection probe is centered at position 550 Kbp on yeast chromosome XIV, so that most recovered molecules should be within position 500–600 Kbp. To confirm this, we generate four qPCR probe sets at different positions on yeast chromosome XIV (Fig. 6f). The yeast genomic DNA is encapsulated in double emulsions and thermal cycled, yielding instances of positive droplets (Fig. 6g). We instruct the instrument to recover these droplets, and extract the DNA templates and analyze them with qPCR (Fig. 6h). We find that probe sets close to the detection probe amplify at low cycles while ones farther away amplify at high cycles (Fig. 6h, inset), indicating that short fragments are more prevalent than long fragments in the sorted sample. Using the qPCR data, we estimate the number of template molecules present in the sample at a given length based on the cross-threshold value of the PCR traces and again find that long molecules are rarer than short molecules but that a significant fraction of long molecules are present, even up to 500 Kbp. This confirms our multiplexed experiment results. In addition, 3dPCR allows molecules above a target length to be recovered by sorting using multiple TaqMan probes separated by this length, which allows recovery of templates hundreds of kilobases in length.

## Conclusions

3dPCR allows DNA molecules to be interrogated and sorted by FACS, providing a novel and powerful means of DNA enrichment. In addition to making the approach more easily accessible than methods based on digital PCR and microfluidic sorting, the use of commercial FACS in 3dPCR affords a number of advantages, including higher throughput, greater measurement sensitivity, and increased probe multiplexing. Previous ddPCR methods were limited to ~1 KHz for droplet sorting^17^ but 3dPCR can sort at rates 5–10x greater. Multiplexing is also valuable, since it allows several distinct sequences to be associated within the same molecule, virus, or cell. While we limit our analysis to DNA, RNA molecules can also be sorted with 3dPCR, only requiring the addition of a reverse transcription step, similar to as has been reported previously for ddPCR. Cells and viruses may also be sorted, although this requires lysis in the double emulsions, so that their nucleic acids are available for the PCR analysis. While detergents present in PCR buffers and the high temperatures for the reaction may be sufficient to lyse many organisms, they may not be for others, such as thermo-tolerant viruses. 3dPCR adds new and powerful capabilities to digital PCR and makes the approach useful for enriching DNA with flow cytometry in a facile method that requires only a microfluidic double emulsion maker. It is useful for studying complex biological systems exhibiting heterogeneity, such as the cell and virus genomes that comprise organismal tissues and tumors, and microbial ecologies inhabiting the human gut.

## Methods

### Reagents and materials

Commercially available HFE, ionic and non-ionic (RAN Biotechnologies) Krytox-based surfactants were used to stabilize the double emulsions for thermal cycling. SU-8 was purchased from MicroChem. The Platinum Multiplex PCR Master Mix (PCR MM) containing dNTPs, PCR buffer, and polymerase, as-well-as the Maxima SYBR Green/Rox qPCR Master Mix (2x) were obtained from ThermoFisher Scientific. Purified Lambda DNA and the ϕX174 Virion DNA were purchased from NewEngland BioLabs. Purified *Saccharomyces cerevisiae* DNA was purchased from Milipore. SYBR Green 1 Nucleic Acid Gel Stain was purchased from Lonza. PolyEthylene Glycol 35 K (PEG35K), PolyEthylene Glycol 6 K (PEG6K), Bovine Serum Albumin (BSA), perfluorooctanol (PFO), and Tween-20 were purchased from Sigma. Pluronic F-68 was purchased from Life-technologies. All oligonucleotides and probes used in this study (Supplemental Table 1) were purchased from Integrated DNA Technologies.

### Device Fabrication

The devices used to make double emulsions were fabricated in PDMS using soft lithography^37^. Planar masters composed of SU-8 were constructed using photolithography and used to mold PDMS devices. Inlet and outlet ports were punched using a 0.75 mm biopsy punch. PDMS devices were bonded to PDMS slabs by treating both with oxygen plasma for 60 s at 1 mbar of pressure in a plasma cleaner. Bonded devices were incubated at 65 °C for 36–48 hours prior to use^38^.

### Oligonucleotides

All oligonucleotides used are listed in Supplemental Table 1.

### SYBR Green PCR

Lambda DNA was diluted in DNAse-free water prior to use. For purification experiments, *S. cerevisiae* DNA was also utilized. PCR reagents were setup as such: 25 μL PCR MM, 4 μL Tween-20 (50%), 4 μL PEG 6 K (50%), 1 μL primer (10 μM), 1 μL primer (10 μM), 1 μL diluted DNA, 14 μL DNAse-free water. Prior to use, the carrier phase inlet port and the emulsion outlet port of the bonded and baked devices were exposed to 1 mbar of oxygen plasma for 55 seconds^38^. Syringes containing (1) PCR mixture, (2) HFE oil supplemented with 2% PEG surfactant, and (3) carrier phase (10% PEG35K, 4% Tween-20, and 1% Pluronic F-68) were attached to their respective inlets via polyethylene tubing. Computer controlled syringe pumps were used to inject fluids at controlled volumetric flow rates (150 μL/hour for PCR mixture, 250 μL/hour for HFE supplemented with surfactant, and 1100 μL/hour for carrier phase) while being monitored visually on a microscope equipped with a short-shutter camera. Double emulsions were collected, supplemented with 15 mM Tris pH 8.0, 40 mM potassium chloride, and 1.5 mM MgCl_2_, and cycled using a Bio-Labs thermal cycler using the following parameters (86 °C for 2 min; 35 × 86 °C for 30 sec, 60 °C for 30 sec, 72 °C for 30 sec; 72 °C for 5 min). After thermal cycling, samples were treated with 1× SYBR Green diluted in DMSO prior to visualization on an EVOS inverted fluorescence microscope.

### TaqMan PCR

Lambda DNA and ϕX174 Virion DNA was diluted in DNAse-free water prior to use. PCR reagents were setup as followed: 25 μL PCR MM, 5 μL BSA (20%), 2.5 μL probe (5 μM), 0.5 μL primer (100 μM), 0.5 μL primer (100 μM), 2 μL diluted DNA, DNAse-free water to 50 μL. Prior to use, the carrier phase inlet port and the emulsion outlet port of the bonded and baked devices were exposed to 1 mbar of oxygen plasma for 55 seconds^38^. Syringes containing (1) PCR mixture, (2) HFE oil supplemented with 2% ionic krytox, and (3) carrier phase (10% PEG35K, 4% Tween-20, and 1% Pluronic F-68) were attached to their respective inlets via polyethylene tubing. Computer controlled syringe pumps were used to inject fluids at controlled volumetric flow rates (150 μL/hour for PCR mixture, 250 μL/hour for HFE supplemented with surfactant, and 1100 μL/hour for carrier phase) while being monitored visually on a microscope equipped with a short-shutter camera. Double emulsions were collected, supplemented with 15 mM Tris pH 8.0, 40 mM potassium chloride, and 1.5 mM MgCl_2_, and cycled using a Bio-Labs thermal cycler using the following parameters (86 °C for 2 min; 35 × 86 °C for 30 sec, 60 °C for 90 sec, 72 °C for 20 sec; 72 °C for 5 min). After thermal cycling, samples were visualized on an EVOS inverted fluorescence microscope.

### Microscopy

Double emulsions were imaged before and after thermal cycling using the EVOS Cell Imaging System from ThermoFisher Scientific. Images were taken under a 10× and 20× objective using the EVOS Cy5 and FITC LED light sources. Images we wanted to compare were all taken using identical illumination settings. After image acquisition, images were commonly overlaid using ImageJ to identify emulsions that contain more than one fluorophore.

### Flow cytometry of 3dPCR reactions

Double emulsions were commonly measured and sorted using the FACSAriaII system from BD. Samples were diluted in a diluent (2% Pluronic F-68, 1% PEG35K) and loaded onto the FACSAriaII via the stage. Double emulsions were first gated using SSC and FSC diffraction, followed by gating using the presence or absence of the SYBR, FAM, or Cy5 fluorophore. SYBR Green and FAM fluorescence were identified using a 488 nm laser and a 505LP optical filter (BD Biosciences). The Cy5 fluorophore was identified using a 633 nm laser with a 670/30 filter. The TaqMan and SYBR experiments produce “binary” signals where a bright fluorescence, or positive reaction, is separated from the dim distribution. An emulsion is defined as positive if it lies within a user-defined fluorescence range. The number of positive double emulsions is recorded by the FACSAriaII and divided by the total number of droplets processed.

### Quantitative PCR

Quantitative (q)PCR was performed using a Stratagene Mx3005P machine. Prior to running, DNA input was normalized to the number of emulsions collected. For identification of (Lambda) targets pre- and post-sorting, primers EP07 and EP08 were used in the qPCR reaction. For indentification of offtarget (*S. cerevisiae*) DNA presence pre- and postsorting, Ye01 and Ye02 were used in the qPCR reaction. In general, cycling conditions used were 95 °C for 5 min, followed by 40× cycles of 95 °C 30 sec, 55 °C 1 min, and 72 °C 30 sec.

## Additional Information

**How to cite this article**: Sukovich, D. J. *et al*. Sequence specific sorting of DNA molecules with FACS using 3dPCR. *Sci. Rep.* **7**, 39385; doi: 10.1038/srep39385 (2017).

**Publisher's note:** Springer Nature remains neutral with regard to jurisdictional claims in published maps and institutional affiliations.

## Supplementary Material

Supplementary Table 1
