# Supplementary material for: Sequence specific sorting of DNA molecules with FACS using 3dPCR
Source: Sci Rep. 2017 Jan 4;7:39385. doi: 10.1038/srep39385 (PMC5209659; doi:10.1038/srep39385)
Supplement: Supplementary Table 1 [file srep39385-s1.pdf]

Sequence specific sorting of DNA molecules with FACS using 3dPCR

David J. Sukovich[1.2], Shea T. Lance[1,2,3], Adam R. Abate[1,2,3,*]

[1]Bioengineering and Therapeutic Sciences, University of California San Francisco, San Francisco, California, USA
[2]California Institute for Quantitative Biosciences, University of California San Francisco, San Francisco, California, USA
[3]UC Berkeley-UCSF Graduate Program in Bioengineering, University of California San Francisco, San Francisco, CA, USA
*Corresponding adam@abatelab.org

Supplemental Table 1

	Sequence (5'-3')	Used for
EP01	GACTACATCCGTGAGGTGAATG	Quantification of Lambda; 2-fluorophore experiments
EP02	TTGCGCTGCTTATGCTCTAT	Quantification of Lambda; 2-fluorophore experiments
EP03	/5Cy5/TCTGCCCGTGTCGGTTATTCCAAA/3IAbRQSp/	Quantification of Lambda; 2-fluorophore experiments
EP04	CTTTGAATGCTGCCCTTCTTC	Quantification of Lambda; enrichment experiment
EP05	CAGATAACCATCTGCGGTGATA	Quantification of Lambda; enrichment experiment
EP06	/56-FAM/ATACTGAGC/ZEN/ACATCAGCAGGACGC/3IABkFQ/	Quantification of Lambda; enrichment experiment
EP07	CGGTAGAGGTAAAGGTGGTAAAG	qPCR of Lambda; enrichment experiment
EP08	CTGATAGCTGGCTTAGTGATACC	qPCR of Lambda; enrichment experiment
PX01	GCGCTCTAATCTCTGGGCAT	Quantification of ϕX174; 2-fluorophore experiments
PX02	CAAAGAAACGCGGCACAGAA	Quantification of ϕX174; 2-fluorophore experiments
PX03	/56-FAM/ATGGAACTG/ZEN/ACCAAACGTCGTTAGGCCA/3IABkFQ/	Quantification of ϕX174; 2-fluorophore experiments
YE01	CGGTAGAGGTAAAGGTGGTAAAG	qPCR of Yeast DNA; enrichment experiment
YE02	CTGATAGCTGGCTTAGTGATACC	qPCR of Yeast DNA; enrichment experiment
YE03	GGATCGGTGGATGAAGAACTAA	Detection of Yeast DNA; size distribution experiment
YE04	CCTCATCGTCGTCATCTGAAA	Detection of Yeast DNA; size distribution experiment
YE05	/5Cy5/AGATGAAGA/TAO/TAATGACGCCCACCCA/3IAbRQSp/	Detection of Yeast DNA; size distribution experiment
YE06	GAGCGATAAGGCATCTGAATCT	Detection of Yeast DNA; size distribution experiment
YE07	CTGGCACTCCAATAGCCATAA	Detection of Yeast DNA; size distribution experiment
YE08	/56-FAM/AGGCATCAT/ZEN/TGGCTAAAGAGAGGCA/3IABkFQ/	Detection of Yeast DNA; size distribution experiment
YE09	GTTGAGAAACCCTCGCTAGTT	Detection of Yeast DNA; size distribution experiment
YE10	GGGCCCTCATCACCTATTTC	Detection of Yeast DNA; size distribution experiment
YE11	/56-FAM/AGAAGGCTT/ZEN/TACCTTAACCGGCACT/3IABkFQ/	Detection of Yeast DNA; size distribution experiment
YE12	ACGTAAAGCTGCGGTTATTATTG	Detection of Yeast DNA; size distribution experiment
YE13	GTTCTTTAGCCCTGGTAGTAGTT	Detection of Yeast DNA; size distribution experiment
YE14	/56-FAM/CCCGCAAGT/ZEN/TGTTGCTCCATTCTT/3IABkFQ/	Detection of Yeast DNA; size distribution experiment
YE15	CCTCTCTGAGATGGGTTCTTTG	Detection of Yeast DNA; size distribution experiment
YE16	ATGGCAGAACCTTACCTTTGT	Detection of Yeast DNA; size distribution experiment
YE17	/56-FAM/AAGTTTGTG/ZEN/AGGGTTGGACAGGCT/3IABkFQ/	Detection of Yeast DNA; size distribution experiment
YE18	CTGGACGAGTTGAGCAGTATAAA	Detection of Yeast DNA; size distribution experiment
YE19	ATCAGGCGACTTGGCAATAA	Detection of Yeast DNA; size distribution experiment
YE20	/56-FAM/ACCTCTGAC/ZEN/AACCCAGACAGCAAT/3IABkFQ/	Detection of Yeast DNA; size distribution experiment
YE21	CATAAGCTGCTTGGTCGATTTG	Detection of Yeast DNA; size distribution experiment
YE22	ATCGACAGCTGCAGTGAATAA	Detection of Yeast DNA; size distribution experiment
YE23	/56-FAM/TGTTGAGAC/ZEN/TGCTGTTGCTCTGCA/3IABkFQ/	Detection of Yeast DNA; size distribution experiment
YE24	TACCAAGGAAACCTGGAATGG	Detection of Yeast DNA; size distribution experiment
YE25	GGGATGGTGATCTCTGTACTTG	Detection of Yeast DNA; size distribution experiment
YE26	/56-FAM/AGCATAGGC/ZEN/GTGCAAGATCCACAT/3IABkFQ/	Detection of Yeast DNA; size distribution experiment
YE27	CCGAACGACACCATAGACATT	Detection of Yeast DNA; size distribution experiment
YE28	CTGAGGATAGCCTCGTTGTTAG	Detection of Yeast DNA; size distribution experiment
YE29	/56-FAM/TTCCAATCT/ZEN/CCGCAACACCTTCGT/3IABkFQ/	Detection of Yeast DNA; size distribution experiment
YE30	TGCGCGAGACTATGCTTTAC	qPCR of Yeast DNA; size distribution experiment
YE31	CCGAGTGACAAAGTACGGTTAG	qPCR of Yeast DNA; size distribution experiment
YE32	CCTCTTCGTCTTCTTCCTCATC	qPCR of Yeast DNA; size distribution experiment
YE33	ACGGATGCTGCTACCATAAA	qPCR of Yeast DNA; size distribution experiment
YE34	CGGATCCATCCTCATCATCTTC	qPCR of Yeast DNA; size distribution experiment
YE35	CCAGAACAGGAACTCCAATACA	qPCR of Yeast DNA; size distribution experiment
YE36	AGCGAAGAGGTTTCTGAGTATG	qPCR of Yeast DNA; size distribution experiment
YE37	GAGGGTGGTTCTGTCGTTTAT	qPCR of Yeast DNA; size distribution experiment
